# Development of Epigenetic Clocks for Key Ruminant Species

**DOI:** 10.3390/genes13010096

**Published:** 2021-12-30

**Authors:** Alex Caulton, Ken G. Dodds, Kathryn M. McRae, Christine Couldrey, Steve Horvath, Shannon M. Clarke

**Affiliations:** 1 AgResearch Limited, Invermay Agricultural Centre, Puddle Alley, Mosgiel 9092, New Zealand; ken.dodds@agresearch.co.nz (K.G.D.); kathryn.mcrae@agresearch.co.nz (K.M.M.); shannon.clarke@agresearch.co.nz (S.M.C.); 2 Department of Biochemistry, University of Otago, Dunedin 9016, New Zealand; 3 Livestock Improvement Corporation, Hamilton 3286, New Zealand; christine.couldrey@lic.co.nz; 4 Department of Human Genetics, David Geffen School of Medicine, University of California Los Angeles, Los Angeles, CA 90095, USA; shorvath@mednet.ucla.edu

**Keywords:** epigenetic clock, methylation, livestock, sheep, goat, deer, cattle

## Abstract

Robust biomarkers of chronological age have been developed in humans and model mammalian species such as rats and mice using DNA methylation data. The concept of these so-called “epigenetic clocks” has emerged from a large body of literature describing the relationship between genome-wide methylation levels and age. Epigenetic clocks exploit this phenomenon and use small panels of differentially methylated cytosine (CpG) sites to make robust predictions of chronological age, independent of tissue type. Here, we present highly accurate livestock epigenetic clocks for which we have used the custom mammalian methylation array “HorvathMammalMethyl40” to construct the first epigenetic clock for domesticated goat (*Capra hircus*), cattle (*Bos taurus*), Red (*Cervus elaphus*) and Wapiti deer (*Cervus canadensis*) and composite-breed sheep (*Ovis aries*). Additionally, we have constructed a ‘farm animal clock’ for all species included in the study, which will allow for robust predictions to be extended to various breeds/strains. The farm animal clock shows similarly high accuracy to the individual species’ clocks (*r* > 0.97), utilizing only 217 CpG sites to estimate age (relative to the maximum lifespan of the species) with a single mathematical model. We hypothesise that the applications of this livestock clock could extend well beyond the scope of chronological age estimates. Many independent studies have demonstrated that a deviation between true age and clock derived molecular age is indicative of past and/or present health (including stress) status. There is, therefore, untapped potential to utilize livestock clocks in breeding programs as a predictor for age-related production traits.

## 1. Introduction

The premise of using biomarkers to predict longevity emerged from an understanding that chronological age is an imperfect representation of the ageing mechanism and a more informative measure is the decline in functional capability with age, that is, an individual’s biological age [1]. In recent years, DNA methylation has been hailed as the most promising marker of biological age [2,3]. Despite the tissue-specific nature of age-associated physiological decline, cytosine methylation correlates strongly with age across virtually all tissue types. This unique characteristic can be exploited in the development of multivariate age estimators (pan-tissue epigenetic clocks) that can accurately predict the age of an individual, independent of the tissue of origin of the DNA sample. For example, the first human pan-tissue clock, which is based on 353 age-related CpGs, estimates chronological age with a correlation of 0.96 and a median error of 3.6 years [4]. Since the development of this clock, epigenetic clocks have been successfully established for model species such as mice. More recently, Lu et al. (2021) constructed three broadly applicable mammalian epigenetic clocks from a large scale metanalysis of methylation data profiled using the HorvathMammalMethyl40 array [4,5]. The clocks show high accuracy (*r* > 0.96) at predicting age across all 142 mammalian species and 57 tissue types assayed in the study, supporting the notion that a universally regulated mechanism underlies the aging process in mammalian species. This provides great potential for the use of an epigenetic clock for livestock breeding purposes. Establishing an accurate biomarker of age would be of use in farming systems where it is not possible to record birth dates with certainty. In such circumstances, age cannot be reliably included in genetic prediction models, which results in reduced selection accuracy.

Perhaps of greater significance is that the estimates generated by this clock and its related counterparts are not only predictive of chronological age, but also biological age, providing a mechanism to infer age-related pathologies and identify genetic, environmental or lifestyle factors that accelerate or slow biological aging [6]. For example, in humans, accelerated epigenetic ageing has been associated with a vast range of disorders from metabolic, infectious, and degenerative disease to frailty, traumatic stress and post-traumatic stress disorder as well as lifestyle factors such as smoking and obesity [7,8,9]. These findings proffer the potential to utilize epigenetic clocks as a biomarker for health and age-related degeneration, a tool that could be deployed in the livestock sector to complement the current genetic framework for the selection of traits such as longevity [10]. More specifically, early selection of animals with a “reduced biological age”, i.e., slower aging, could result in animals with improved longevity. An incorporation of the “biological age” in genetic evaluations to adjust an individual’s performance may promote better selection decisions [11]. Alternatively, epigenetically younger animals may have a preference for retainment in the herd as a reflection of greater resilience. Furthermore, Lu et al. (2021) have shown that maximum lifespan (the genetic limit of longevity in an ideal environment) can also be predicted via methylation-based models [12]. Utilising the epigenetic clock as a biomarker in livestock breeding schemes would provide an objective predictor of stayability and health measures.

In New Zealand, the primary sector is dominated by the dairy cattle, beef cattle and sheep industries. In 2020 the number of beef cattle was 4 million, with 6.1 million dairy cattle, and 26.2 million sheep. The deer industry, comprised mainly of Red and Wapiti breeds, is well established for venison and velvet production, and total deer numbers approach 1 million. While goat farming has a lower profile, dairy goats are gaining popularity for their unique milk properties that are favourable for human infant formula, and currently, dairy goats number approximately 70,000. Breeding programs are well established nationally across these key species, providing a strong foundation for the industry to capitalise on novel approaches to enhance the prevailing genetic merit predictions. Towards this goal, we have constructed an epigenetic clock for livestock species that are particularly relevant to New Zealand’s agricultural interests to accurately estimate the chronological age of sheep, goat, deer and cattle (*r* = 0.97), which also has the potential to be extended to closely related arteriodactyls. This work provides a tool to determine the biological age of livestock, which will be particularly useful in farm systems and has potential as a molecular phenotype for stress resilience and age-related degeneration for breeding purposes. 

## 2. Materials and Methods

### 2.1. Deer Samples

Although Red deer (*C. elaphus*) and Wapiti deer (*C. canadensis*) are classified as separate species, they are capable of interbreeding and producing fertile offspring. In the New Zealand deer industry, Red and Wapiti are considered different breeds rather than different species, thus, to maintain consistency we will also refer to them as breeds and treat them as a single group for clock development [13]. Ear tissue punches (Allflex Tissue Sampling Unit; TSU samples) of deer were obtained from Focus Genetics, Napier, New Zealand. The animals are a mix of crossbred and purebred Red and Wapiti breeds that were sampled from four different studs. While individuals in the dataset are not necessarily purebred, they are represented by their predominant breed. Chronological ages ranged from 1 month to 13 years and 8 months old. A total of 74 hinds and 22 stags were included in the dataset. Comprehensive birth date and sample date records allowed for the age of the animal at the time of sampling to be determined to the nearest day.

### 2.2. Cattle Samples

TSU samples of cattle (*B. taurus*) were obtained from Livestock Improvement Corporation (LIC) Ltd., Hamilton, New Zealand. The animals were sampled from LIC’s research dairy herd. The majority were Kiwicross breed (Jersey/Friesian cross) with some full Jersey breed and some full Friesian breed included in the dataset. All 96 samples were female. The age range was from 1 month to 11 years and 1 month old, the age of the animal at the time of sampling was determined to the nearest day.

### 2.3. Goat Samples

TSU samples from one flock of Saanen breed dairy goats (*C. hircus*) were obtained from New Zealand goat breeders, Judy and Barry Foote, Hikurangi, New Zealand. The dataset was made up of 72 does, 23 bucks and 1 intersex individual (visible male sex organs in addition to a milk-producing udder). Ages ranged from 2 weeks to 8 years and 7 months of age, determined to the nearest fortnight.

### 2.4. Sheep Samples

TSU samples were obtained from two research sheep flocks (*O. aries*) from AgResearch Ltd., Woodlands, NZ. The sheep were a composite breed resulting from the cross of Coopworth (~54%), Texel (~18%), Romney (~12%), and East Friesian (~10%) and other breeds (~6%). The age of the animals at the time of sampling ranged from 16 days to 7 years and 1 month old, determined to the nearest month. There were 73 ewes and 23 rams included in the dataset.

### 2.5. Life History Traits

Life-history traits for the four species, including maximum lifespan, average age at sexual maturity and average gestation length were sourced from the Animal Aging and Longevity Database (AnAge [14], http://genomics.senescence.info/help.html#anage, accessed on 24 June 2021), (Table 1). This was to ensure consistency across clock development in livestock and other mammals [15,16,17,18]. While the AnAge estimates for age at sexual maturity and gestation length are outside of what is recorded in New Zealand farming systems, there is little effect on the clock predictor as they are proportionally comparable across species.

### 2.6. Extraction and Bisulphite Conversion of DNA

Ear punch tissue was collected using TSUs, and genomic DNA was extracted from tissue samples in 96-well plates using a high-salt method that has been shown to yield good quality, high molecular weight DNA [19]. Extraction and subsequent normalisation were performed with automated liquid handling robots.

Stock DNA was quantified using a Nanodrop8000 (Thermo Fisher Scientific Inc, Waltham, MA, USA). The DNA was then diluted in autoclaved MilliQ water and normalised to approximately 80 ng/µL in a 96-well plate. The normalised DNA plates were then quantified using an intercalating dye (PicoGreen; Thermo Fisher Scientific Inc, Waltham, MA, USA) and an automated VICTOR3 fluorometer (Perkin Elmer, Inc., Waltham, MA, USA) [20]. Bisulphite conversion of 1 µg of input DNA was carried out with EZ DNA Methylation-Gold Kit (Zymo Research, Waltham, MA, USA) following the manufacturer’s instructions.

### 2.7. Infinium Array

A custom Illumina methylation array “HorvathMammalMethyl40” (Illumina, Inc., San Diego, CA, USA; [5]) was used to measure methylation for up to 37,000 CpG sites, selected for their highly conserved flanking sequences across the Mammalia class. Array hybridisation and staining were performed with an automated liquid handling robot (Tecan Trading AG, Männedorf, Switzerland), and array scanning and imaging were executed on the Illumina Iscan platform (Illumina, Inc., San Diego, CA, USA).

### 2.8. Data Pre-Processing

Methylation levels are reported as *β*-values calculated using the formula:

(1) $$\beta =\frac{M}{M+U+a}$$

where *M* and *U* represent the methylated and unmethylated signal intensities (positive values), respectively. The offset *a* ≥ 0, typically set to 100, was added to *M + U* to stabilize *β* values when both *M* and *U* are small [21]. The SeSAMe analysis suite was used to generate normalised *β* values for each probe on a scale between zero (completely unmethylated) and one (completely methylated) from the raw data files [22]. SeSAMe assesses the efficiency of probe hybridisation and extension using Infinium-I probe out-of-band measurements (the pOOBAH method) and effectively masks spurious methylation calls that result from incomplete probe hybridisation [22]. SeSAMe has previously been implemented for the normalisation of the Mammalian Methylation array and was shown to slightly outperform a similar R package minfi, which implements the normal-exponential convolution using out-of-band probes (NOOB) method of normalisation [5,23].

Hierarchical clustering of the samples for each species was performed with the R package WGCNA [24] to establish if there was any underlying structure in the datasets that could be attributed to the known covariates, including array batch effects or breed variation. Six sheep samples were excluded by quality control measures due to suspected contamination when their sex check results were nonconclusive, i.e., the samples clustered more closely with samples of the opposite sex to their recorded sex.

### 2.9. Age Transformations for Clock Construction

In line with the universal mammalian clock development, we constructed three epigenetic clocks per species based on three different transformations of age, two of which allowed for comparisons to be made across different species (with different lifespans) for the purpose of constructing multi-species’ clocks: (1) log-transformed chronological age; (2) −log(−log(*RelativeAge*)); and (3) log-linear transformed age [12]. Epigenetic age estimates of each clock were computed via the inverse. The age transformations used to build clocks 1 to 3 incorporated three life-history trait measurements—gestational time (*GT*), age at sexual maturity (*ASM*), and maximum lifespan (*MaxAge*), sourced from the AnAge database and measured in units of days (Table 1).

#### 2.9.1. Log Transformed Chronological Age (Clock 1) Transformation

Clock 1 correlates the epigenetic age with the observed chronological age. For the log transformation of chronological age (to *LogAge*), an age offset of 2 times gestational time was added which allows for the ages of prenatal samples to be used in future applications and ensures that the log transformed chronological age (Formula (2)) does not change as dramatically at very young ages. This is also consistent with the idea that the epigenetic clock begins “ticking” from the moment of conception. The log transformation and the inverse transformation (to *DNAmAge*) are:

(2) LogAge=log(Age+2×GT)

(3) DNAmAge=exp(LogAge)−2×GT

#### 2.9.2. Loglog Transformation of Relative Age (Clock 2)

Clock 2 determines the relative age of an animal and was developed to allow for biologically meaningful comparisons to be made between the four species by accounting for their differing maximum lifespans. Clock 2 leverages both gestational time and maximum lifespan to determine age relative to maximum age on a scale between 0 and 1. Relative age was defined as 2 times the gestational time, for similar reasons as clock 1, and maximum lifespan as follows:

(4) $$RelativeAge=\frac{Age+2\times GT}{MaxAge+2\times GT}\;$$

(5) LoglogAge=−log(−log(RelativeAge))

Clock 2 employs *LoglogAge* and subsequently applies the inverse transformation to predict epigenetic age:

(6) DNAmAge=exp(−exp(−LoglogAge))×(maxAge+2×GT)−2×GT

#### 2.9.3. Log-Linear Transformation of Age (Clock 3)

Clock 3 leverages age at sexual maturity as a proxy for a species’ maximum lifespan as it correlates strongly with maximum lifespan on the log scale (Pearson correlation *r* = 0.82, *P* = 6 × 10^−183^ across all mammalian species in AnAge [12]). Age at sexual maturity is often better characterised in animals compared to maximum lifespan, particularly in livestock that are not typically retained beyond their productive lifespan.

Clock 3′s age transformation takes the logarithmic form when age is less than *ASM* and takes the linear form when age is greater than *ASM* and is continuously differentiable at *ASM*. This model accommodates faster changes in methylation through development.

First, we calculate *RelativeAdultAge* as a ratio of age to *ASM* where an offset of 2 times the gestational time is added, as in the first two clocks.

(7) $$RelativeAdultAge=\frac{Age+2\times GT}{ASM+2\times GT}$$

The ‘*Log-linear age*’ is then defined as:

(8) $$LoglinearAge=\{\begin{array}{c} RelativeAdultAge-1,\;RelativeAdultAge\geq 1\\ \log\left(RelativeAdultAge\right),\;RelativeAdultAge<1 \end{array}$$

Clock 3 predicts *LoglinearAge* and applies the inverse transformation to estimate epigenetic age as follows.

(9) $$\begin{array}{c} DNAmAge\\ =\{\begin{array}{c} ASM+LoglinearAge\times \left(ASM+2\times GT\right),\;LoglinearAge\geq 0\\ \exp\left(LoglinearAge\right)\times \left(ASM+2\times GT\right)-1.5,\;LoglinearAge<0 \end{array} \end{array}$$

### 2.10. Elastic Net Regression

To build epigenetic clocks for each species, an elastic-net regression model was implemented using the R package glmnet [25] within R v 3.626 (R Foundation for Statistical Computing, Vienna, Austria) [26]. Elastic net regression combines the regularisation of both ridge regression and LASSO (least absolute shrinkage and selection) regression, meaning that it is suited to high dimensional datasets where the number of predictors is more than the number of observations and where predictors are likely to be highly correlated [25]. As part of the model, a subset of features (CpG methylation sites) are selected which give the best predictor for a set of outcomes (age). The two main parameters that are employed in the elastic net model are: (1) the mixing parameter (α) which controls the shrinkage type between ridge (α = 0) and LASSO regression (α = 1) and; (2) the penalty parameter (λ) which controls the stringency of penalty (higher values of λ lead to coefficients closer or equal to zero) [27].

The elastic net parameter α was set to 0.5 as the midpoint between both LASSO and Ridge regularization. The penalty parameter (λ) used was lambda.min (the minimum mean cross-validation error) calculated by the function cv.glmnet through a 10-fold internal cross-validation [28]. The mean error was calculated for each iteration of the cross-validation and then averaged over the ten partitions to determine the λ value that achieved the minimum mean error [25].

The accuracy of the estimators was calculated using leave-one-out cross-validation (LOOCV) by executing the cv.glmnet function for each set of *n*−1 samples, where *n* is the number of samples. The predicted age of the omitted sample was calculated with the model built using data from the remaining samples.

### 2.11. Farm Animal Clock

For the multi-species farm animal clock, we combined all data from all species and applied elastic net regression to the *LoglogAge* = −log(−log(RelativeAge)) as described in Formulas (4)–(6) to construct farm clock 2 and the *loglinearAge* transformation as described in Formulas (7)–(9) to construct farm clock 3. Clock 1, which uses chronological age (rather than relative age) is not recommended for multi-species’ clock development where there is variability in the maximum lifespan of the included species. To assess the accuracy of the farm clocks, we used LOOCV and leave-one-species-out (LOSO) cross-validation. The LOSO cross-validation initially trained the model based on all-but-one species. The model was then tested on the omitted species to assess its generalizability to the species that were not included in the training dataset.

### 2.12. Assessing the Model Prediction Performance

To validate the accuracy of the models, we determined the Pearson’s correlation and calculated the median absolute difference (median absolute error; MAE) between the epigenetic age estimates from LOOCV analysis and the observed age for all samples. The within fold median correlations (med.corr) and MAE as well as the correlations for all folds were computed for the LOSO-based approach for each species. The correlations were calculated using the transformed data (where the relationship was linear).

## 3. Results

### 3.1. Structure of the Raw Data

**Figure 1:**
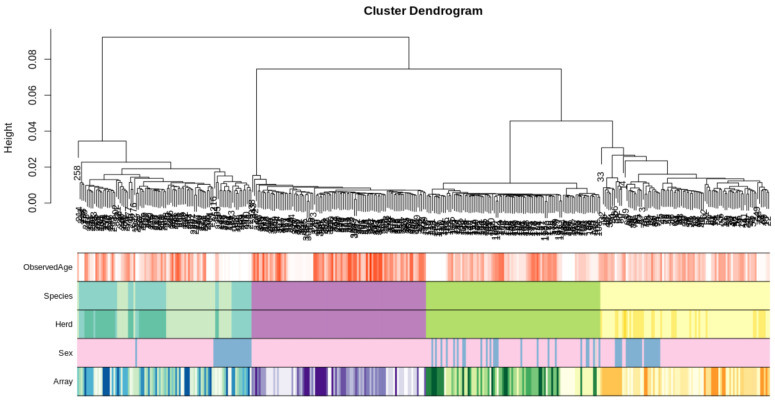
Hierarchical clustering of the complete methylation dataset for each sample (excluding sheep samples that failed QC). Species are coloured yellow for sheep-related covariates, green for goat-related covariates, purple for cattle related covariates and turquoise/blue for deer-related covariates (note: the two shades of turquoise in the species panel denote animals that are predominantly Red (greenish) and Wapiti (blueish) breeds, respectively). Sex is coloured pink for females, blue for males and grey for hermaphrodite samples. The data form four distinct clusters based on species; there is some sub-clustering based on sex; there is no obvious sub-clustering attributed to the array the sample was run on.

Initially, to characterise any substructure in the datasets we performed hierarchical clustering using the full methylation array information for each sample. There were four distinct clusters based on species with moderate sub clustering based on sex, importantly there was no obvious sub-clustering based on batch effects from samples run on the same chip array (Figure 1).

### 3.2. Development of Species-Specific Clocks

The number of CpG sites selected by elastic net regression to construct the models for each species ranged from 45 to 123 sites (Table 2). On average, 60% of the sites for each clock had methylation signals that were negatively correlated and 40% had methylation signals that were positively correlated with age, respectively (Table 2).

### 3.3. Predictive Performance of the Epigenetic Clocks

**Figure 2:**
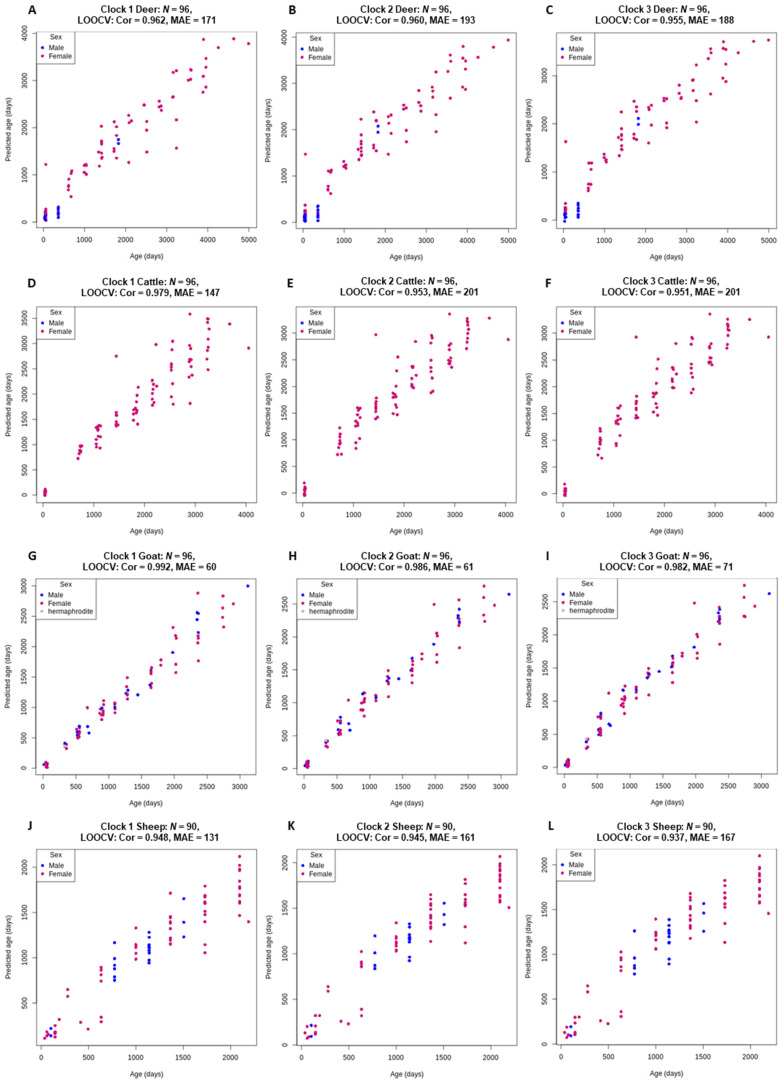
Chronological age (x-axis) versus back transformed epigenetic age estimated using clock 1 (log transformed chronological age; (**A**,**D**,**G**,**J**)), clock 2 (loglogRelativeAge; (**B**,**E**,**H**,**K**)) and clock 3 (log-linear transformed age; (**C**,**F**,**I**,**L**)). For all four species, deer (**A**–**C**), cattle, (**D**–**F**), goat (**G**–**I**) and sheep (**J**–**L**). Pearson’s correlation coefficient estimates (Cor) and median absolute error (MAE) are reported for the age estimates via LOOCV.

All three clocks for each species were highly accurate (*r* > 0.937) with an MAE of less than 7 months (Figure 2). Clock 1 slightly, but consistently, outperformed both clock 2 and clock 3. Goat clock 1 was the most accurate (*r* = 0.992) using only 86 CpG sites for predictions (Table 2). The high accuracy of this clock is likely due to the controlled environment in which the goats were raised and their origination from a single, closed herd.

**Figure 3:**
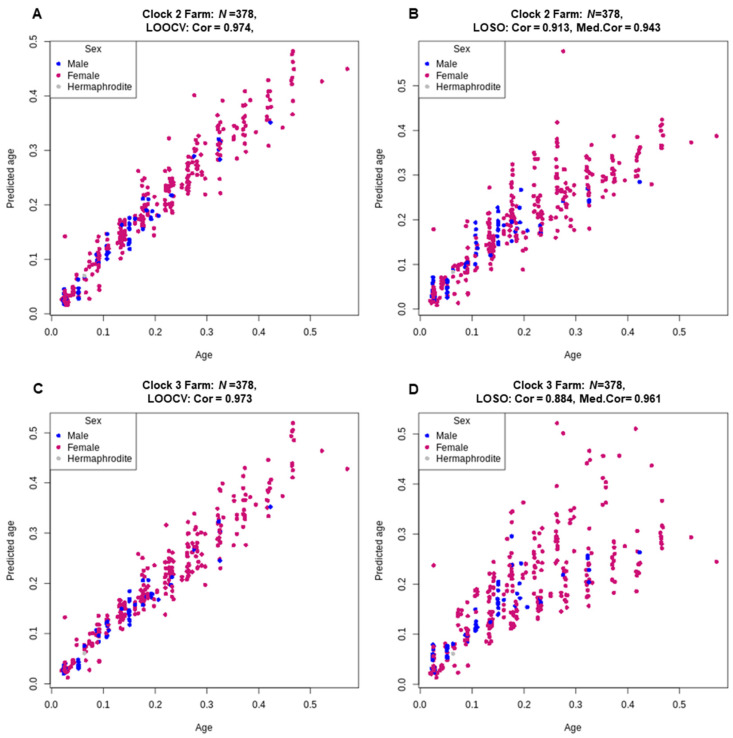
Relative age (x-axis) versus back-transformed relative epigenetic age estimated for Farm clock 2 (loglogRelativeAge; (**A**,**B**)) and Farm clock 3 (log-linear transformed age; (**C**,**D**)). Age estimates were predicted via both LOOCV (**A**,**C**) and LOSO (**B**,**D**) cross-validation. The Pearson correlation coefficients (Cor) are reported for the age estimates using both LOOCV and LOSO cross-validation and the median correlation coefficients (Med.Cor) for each fold of the LOSO cross-validation are reported.

To improve the robustness of the individual clocks, we built a multi-species’ clock using the complete dataset of all four species. For this farm animal clock, we performed both an LOOCV and a LOSO cross-validation to assess its performance. The performance of farm animal clock 2 showed high accuracy, similar to the individual species clocks using 217 CpG sites, with a correlation of 0.97 for the LOOCV cross-validation and more notably, a within species median correlation of 0.94 and across all species a correlation of 0.913 from the LOSO analysis. Clock 3 had a slightly higher accuracy within species median correlation of 0.961 but did not perform as well across species with a slightly lower correlation of 0.884 (Figure 3 and Supplementary Table S1).

### 3.4. Overlap between CpG Sites

**Figure 4:**
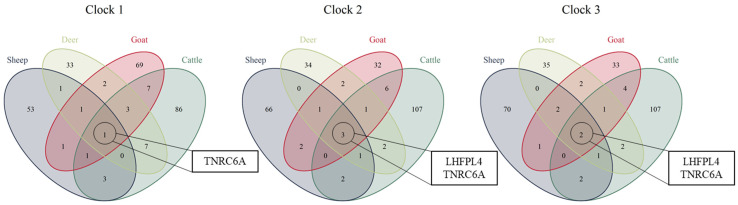
Venn diagrams depicting the overlap of CpG sites included in each of the individual species’ clocks for clock 1, clock 2 and clock 3. The CpG sites common across all four species lie within the genes *TNRC6A* and *LHFPL4*.

There was very little overlap between the CpG sites selected for the clocks of each species. Only three CpG sites were shared across all four species in clock 2, one of these was also commonly shared across species in clock 1 and two were commonly shared across species in clock 3; all three sites involved are located in two functionally annotated genic regions, *TNRC6A* and *LHFPL4* (Figure 4).

The gene *TNRC6A* encodes a member of the trinucleotide repeat containing 6 protein family and was consistently demethylated with increasing age. The protein functions in post-transcriptional gene silencing through RNA interference (RNAi) and microRNA pathways [29]. The demethylation of this gene has previously been linked to ageing in similar epigenetic clock studies in prairie voles [30] and primates [31]. The *LHFPL4* gene is a member of the lipoma HMGIC fusion partner (LHFP) gene family, which is a subset of the superfamily of tetraspan transmembrane protein-encoding genes. Additionally, *LHFPL4* plays a role in the regulation of inhibitory synapse formation and maintaining GABA receptors [32]. While there are no obvious links between its function and the ageing process, two cytosines located in exon 2 of in *LHFPL4* were the most predictive across all species in the development of the recent universal mammalian epigenetic clock and showed a correlation with age of >0.8 in 24 species, implying there is a true although, as of yet unclear function of this gene and its role in the ageing process [12]. Further work by Lu et al., 2021 revealed that methylation of *LHFPL4* cg12841266 was strongly correlated with both development (*r* = 0.58 and *P* = 8.9 × 10^−11^) and post-development stages (*r* = 0.45 and *P* = 2.3 × 10^−76^) across a range of tissue types [12]. Importantly the observed changes in methylation levels of *LHFPL4* were consistently observed to be associated with ageing in young animals as well as both middle-aged and older animals, suggesting that this gene plays a role throughout the lifespan of mammals from development through to adulthood [12].

## 4. Discussion

In the livestock sector, the longevity of an animal is of significant economic importance and also represents a measure of animal welfare and sustainability within the sector [33]. Extending the productive lifespan of an animal increases the profitability of production sectors by (1) reducing the cost of replacement animals; (2) improving the average herd yields by increasing the proportion of animals in higher-producing age groups, for example, a larger proportion of mature cows in dairy cattle schemes; (3) optimising land usage by reducing the acreage required to rear replacement animals; and (4) decreasing involuntary culling (culling productive, profitable animals due to illness, injury, infertility) while increasing voluntary replacement (culling or selling animals that are healthy but that do not meet productivity requirements) [34].

Selection, based on longevity, is complex, as the true lifespan of an animal is only discernible at the end of its natural life, while breeding decisions and culling occur earlier. Longevity or “stayability” is used to reflect the capability of an animal to remain in the herd over time, avoiding both natural attrition and culling. Identifying biomarkers that are predictive of an animal’s functional stayability would greatly enhance the current selection framework for this complex trait. Epigenetic clocks, which are predictive of age-related degeneration in mice and humans, could be an appropriate biomarker towards this objective.

This paper outlined the novel development of individual clocks for four key species of agricultural importance, namely, sheep, goat, deer, and cattle, in addition to a multi-species farm animal clock which is predictive across all four animal groups. The individual species clocks would be expected to be biased towards the animals used to construct their clocks. Therefore, caution is needed in extrapolation to differing breeds or farming conditions. However, the remarkable predictive accuracy of LOSO analysis demonstrates that the multi-species farm animal clock is highly robust and likely to perform well even in some species that are not incorporated in the training dataset and/or in different environments, for example in ruminant livestock species originating from different countries and grazed under differing conditions.

The clocks, developed using three different transformations of age, are relevant across a range of applications. Based on the cross-validation analyses, in general, we suggest using the species-specific clock 1 predictors when estimating the age of animals that are of similar breed composition to those used to build the models presented here. Clock 1, which estimates chronological age rather than relative age (as for clock 2 and clock 3) is the most accurate across the individual species. For all other situations where the clock is to be used to predict the age of sheep, goat, deer, and cattle of distantly related breeds as well as other ruminants, we suggest the use of the multi-species’ clock 2, which demonstrated the most robust predictions across species.

The multi-species farm-animal epigenetic clock 2, represents a tool to accurately estimate the chronological age of sheep, goat, deer and cattle with high accuracy (*r* > 0.97), with promising application to additional livestock species. The results from this work echo the key findings of the universal mammalian epigenetic clock that was trained on a large-scale dataset across 128 mammalian species [12]. Firstly, and most noteworthy, is the robustness of both the farm and universal clocks at predicting biological age across species that were not part of the training set, which reinforces the notion that a highly conserved and defined mechanism underlies biological ageing. Secondly, the *LHFPL4* gene, which is highly predictive of age in both the universal mammalian clocks and the farm animal clocks, appears to play a key role in the ageing process throughout an individual’s lifespan, despite its current functional annotation providing little connection to biological degeneration. The lack of overlap between the CpG sites selected for the clocks of each species can likely be explained by the LASSO element of elastic net regression whereby if there are grouped variables (highly correlated between each other) LASSO tends to select one variable from each group ignoring the others [35]. Therefore, if there are two or more correlated predictors, one may be selected for a specific clock, while it may be reduced to zero in a different clock.

We expect that the livestock clock will prove to be a useful tool for the livestock industry in order to make accurate predictions of biological age. We believe that the clock sites could be incorporated into a small-panel targeted assay to reduce the costs associated with array-based DNA methylation profiling. This study provides a tool to determine the biological age of livestock with great potential to act as a molecular phenotype for age-related degeneration, and stayability traits for breeding purposes.

## Figures and Tables

**Table 1:** Maximum life span and average life-history periods for deer, cattle, goat and sheep sourced from the AnAge database (http://genomics.senescence.info/help.html#anage, accessed on 24 June 2021).

Species	Maximum Lifespan (Years)	Age at Sexual Maturity (Days)	Gestation Length (Days)
Deer	31.5	791	245
Cattle	20	548	277
Goat	20.8	545.5	155
Sheep	22.8	731	146

**Table 2:** Number of CpG sites selected by elastic net regression for the construction of each epigenetic clock.

Clock Type ^1^	Deer Clock			Cattle Clock			Goat Clock			Sheep Clock			Farm Clock	
Age transformation clock number ^1^	1	2	3	1	2	3	1	2	3	1	2	3	2	3
CpG sites with methylation level positively correlated with age	33	27	28	67	67	59	58	37	32	37	43	45	119	146
CpG sites with methylation level negatively correlated with age	16	18	18	42	56	61	28	11	14	25	33	34	98	131
Total number of CpG sites in clock	49	45	46	109	123	120	86	48	46	62	76	79	217	277

^1^ Clock 1 = log transformed chronological age; clock 2 = loglogRelativeAge; and clock 3 = log-linear transformed age.

## Data Availability

The animal data used in this study are the property of the flock and herd owners, thus the information is accessible only with their permission.

## Supplementary Materials

The following are available online at https://www.mdpi.com/article/10.3390/genes13010096/s1, Table S1: The Pearson correlation coefficients for the age estimates via LOSO cross-validation for each fold drop for clock 2 and clock 3. The data is trained on 3 species and tested on the omitted species. For each iteration, we report the correlation between the transformed epigenetic age and the transformed age of the omitted species.
