# Supplementary material for: Development of Epigenetic Clocks for Key Ruminant Species
Source: Genes (Basel). 2021 Dec 30;13(1):96. doi: 10.3390/genes13010096 (PMC8775075; doi:10.3390/genes13010096)
Supplement: Supplementary file 1 [file genes-13-00096-s001.zip › genes-1525682-supplementary.pdf]

Supplementary Material

Table S1. The Pearson correlation coefficients for the age estimates via LOSO cross-validation for each fold drop for clock 2 and clock 3. The data is trained on 3 species and tested on the omitted species. For each iteration we report the correlation between the transformed epigenetic age and the transformed age of the omitted species.

Test species	LOSO CV correlation (Clock 2)	LOSO CV correlation (Clock 3)
**Deer**	0.93	0.84
**Cattle**	0.96	0.95
**Goat**	0.98	0.92
**Sheep**	0.92	0.89
